# A phase I study of the safety and activity of K-001 in patients with advanced pancreatic ductal adenocarcinoma

**DOI:** 10.1186/s12885-021-08375-6

**Published:** 2021-06-07

**Authors:** Jiujie Cui, Haiyan Yang, Jue Liu, Donghui Chen, Jiong Hu, Haiyan Zhang, Yu Wang, Ting Han, Tiebo Mao, Feng Jiao, Ewelina Biskup, Yaotian Pan, Min Liu, Liwei Wang

**Affiliations:** 1 Department of Medical Oncology, Renji Hospital, School of Medicine, Shanghai Jiao Tong University, Shanghai, China; 2 State Key Laboratory of Oncogene and Related Genes, Shanghai Cancer Institute, Renji Hospital, School of Medicine, Shanghai Jiao Tong University, Shanghai, China; 3 Department of Oncology, First People’s Hospital, Shanghai Jiaotong University, Shanghai, China; 4 Department of Epidemiology and Biostatistics, School of Public Health, Peking University, Beijing, China; 5 College of Fundamental Medicine, Shanghai University of Medicine and Health Sciences, Shanghai, China; 6 Institute of Psychology Chinese Academy of Sciences, Beijing, China

**Keywords:** Pancreatic ductal adenocarcinoma, Phase I study, Safety, K-001, Clinical trial

## Abstract

**Background:**

Pancreatic ductal adenocarcinoma (PDAC) is a deadly disease that lack of effective therapeutic drugs. K-001 is an oral antitumor drug made from active ingredients of marine microorganisms. The current study aimed to evaluate safety and antitumor activity of K-001 in patients with advanced PDAC.

**Methods:**

In this phase I, open-label trial, patients with advanced PDAC were recruited to a dose-escalation study in a standard 3 + 3 design. K-001 was administered twice daily in four-week cycles, and dose escalation from 1350 mg to 2160 mg was evaluated twice daily. Physical examination and laboratory tests were done at screening and then weekly. The safety, dose-limiting toxicity (DLT), and maximum tolerated dose (MTD) of K-001 were assessed while tumor response was estimated by Response Evaluation Criteria in Solid Tumor (RECIST).

**Results:**

Eighteen patients with advanced PDAC were screened, and twelve eligible patients were analyzed in the study. No DLT was observed. Totally, 47 adverse events (AEs) presented, and 14 drug-related AEs were reported in 7 patients, including 8 grade 1 events (57.1%) and 6 grade 2 events (42.9%). There was no grade 3 or 4 drug-related AE. In these 14 drug-related AEs, the most frequent ones were dyspepsia (21.4%), followed by flatulence, constipation, and hemorrhoid bleeding (above 10% of each). Among all 12 patients, 10 patients (83.3%) maintained stable disease (SD), and 2 patients (16.7%) had progressive disease (PD). The objective response rate (ORR) was 0% and the disease control rate (DCR) was 83.3%.

**Conclusions:**

K-001 manifests satisfactory safety and tolerability, as well as meaningful antitumor activity in advanced PDAC patients. Further evaluation of K-001 in phase II/III appears warranted.

**Trial registration:**

NCT02720666. Registered 28 Match 2016 - Retrospectively registered.

**Supplementary Information:**

The online version contains supplementary material available at 10.1186/s12885-021-08375-6.

## Background

Pancreatic ductal adenocarcinoma (PDAC), also called pancreatic cancer, is among the most lethal cancer types world-wide with high mortality that almost closely parallels incidence, and is chemoresistant with no more than 30% response rate to standard treatment [1]. Most PDAC patients who accept surgery will relapse within one or two years. Although the risk factors and genomic profile of cancers and PDAC have been widely investigated, the molecular pathogenesis of PDAC remains unclear, and it is difficult to early diagnose PDAC [2–7]. The 5-year survival rate of PDAC is less than 7% (the data has not fluctuated significantly in the past 20 years), and it has been estimated that PDAC may emerge as the second leading cause of cancer-related deaths by 2030 [8]. Particularly, about 85% of PDAC patients have already developed into incurable metastatic or locally advanced stage at the time of diagnosis [9]. Thus, drug-based comprehensive treatment is essential for PDAC patients, but the options are quite limited.

Compared with the rapid development of therapies for other types of cancer, the options of treatment, including drugs, radiotherapy and etc. for PDAC have been lacking in clinical progress and the adverse reactions were not acceptable for some PDAC patients [10]. Targeting and immunological therapies have shown efficacy or promise for certain types of cancer, but have not yet achieved similar results for pancreatic cancer [11–14]. Gemcitabine (GEM) has replaced fluorouracil as the standard first-line treatment since 1997, with the primary endpoint of “clinical benefit responses” including measurements of pain, performance status, and weight [15]. The tolerance of GEM was quite well, but the efficacy was unsatisfactory with 5.65 months of median overall survival (OS) and 18% of the 1-year OS rate. Thereafter, many combinations of GEM with a variety of cytotoxic and targeted agents have been investigated, but no added benefit was observed in OS [16–21]. In 2007, Erlotinib plus GEM got a positive result statistically, but the median OS was only 6.24 months in combination group as compared with 5.91 months in GEM group [22]. In 2011, a phase III study showed that irinotecan, oxaliplatin, and leucovorin-modulated fluorouracil (FOLFIRINOX) significantly improved the median OS compared to GEM (11.1 months vs 6.8 months) [23]. But the toxicities, such as neutropenia, diarrhea, and peripheral neuropathy were also significantly increased in FOLFIRINOX group, limiting the widespread use of FOLFIRINOX in Asian or patients with poor performance status. In 2013, a phase III trial in Japan and Taiwan showed that S-1 was not inferior to GEM in median OS [24]. Another blockbuster study showed that nab-paclitaxel plus GEM significantly improved the median OS and progression-free survival (PFS), with acceptable tolerance [25]. For second line therapy, nanoliposomal irinotecan in combination with fluorouracil and folinic acid prolonged the median OS, but the nanoliposomal irinotecan was unlisted in China, and the Chinses PDAC patients are lack of standard second and later lines of therapy [26]. Clinical studies in recent years have exhibited some degree of progress on treatment of PDAC, nonetheless, toxic effects and tolerance are still the major concern in the treatments, especially for patients of several line therapies, or patients with poor performance status, who are intolerable to mono or multiple chemotherapy.

It is obvious that a safe, effective, and low toxic drug is highly demanded in PDAC treatment. It has been reported that antitumor drugs screened from natural products are safer and lower toxic than that from synthetic drugs [27]. K-001 is a biological compound made from active ingredients of marine microorganisms. K-001 was one of the 1.1 classes novel drugs of China, and was developed by Beijing Hwealth Bio-Pharmaceutical Co. Ltd. The main components of K-001 are peptidoglycan (PGN) and its molecular weighs more than 100,000 Da. The preclinical observation and previously clinical study showed that K-001 was a non-toxic or slightly toxic substance. In the previous phase I study of K-001 among multiple kinds of advanced refractory solid tumors, four doses were tested (670 mg, 1350 mg, 2025 mg and 2700 mg daily), and dose limited toxicity (DLT) was not observed. The adverse event (AEs) of this phase I study were relative few which indicated that the toxicity of K-001 was quite low. Moreover, the study showed that K-001 could improve performance status, appetite and quality of life, which are also highly demanded for PDAC. Based on those prior studies, we conducted the current study which focused on the safety and antitumor activity of oral drug K-001 in patients with advanced PDAC. The study used the standard 3 + 3 design, and four escalating dose levels were included in the trial. 12 patients completed the trial and 47 AEs were observed and no DLT of oral K-001 was observed. According to the RECIST 1.0 criteria, the objective response rate (ORR) was 0% and the 4-week disease control rate (DCR) was 83.3%.

## Methods

The current study was a phase I, open-label, single-center, dose-escalation clinical trial to determine the dose limited toxicity (DLT), the maximum tolerated dose (MTD) and the recommended dose (RD) for phase II/III trials, as well as the preliminary antitumor effects of K-001 in patients with advanced PDAC. The study was registered with the US National Library of Medicine (ClinicalTrials.gov) as NCT02720666.

### Patient eligibility criteria

The following inclusion criteria were used for participant selection: (1) aged 18–70 years; (2) histologically or cytologically confirmed locally advanced or metastatic PDAC; (3) relapsed or refractory to standard therapy; (4) above 28 days from the end of the last chemotherapy; (5) unsuitable or unwilling to standard therapy; (6) at least one measurable or assessable target lesion as defined by the Response Evaluation Criteria In Solid Tumors version 1.0 (RECIST v1.0) [28]; (7) the Eastern Cooperative Oncology Group performance status (ECOG PS) ≤1; (8) life expectancy of longer than three months; (9) ability to take medications orally; (10) hematopoietic function (absolute neutrophils count ≥1.5 × 10^9^ /L, hemoglobin≥9.0 g/dL, platelets count ≥80 × 10^9^ /L); (11) hepatic function (bilirubin ≤1.5 × upper limit of normal (ULN), albumin≥3.0 g/dL, alanine aminotransferase (ALT) and aspartate aminotransferase (AST) ≤ 3.0 × ULN in patients without liver metastasis, or ALT and AST ≤ 5.0 × ULN in patients with liver metastasis); (12) renal function (serum creatinine ≤1.5 × ULN, creatinine clearance rate ≥ 60 ml/min).

Patients were excluded on criteria of (1) non-adenocarcinoma of pancreatic tumors; (2) the target lesion had been treated by radiotherapy with no progression prior to the current trial; (3) central nervous system or leptomeningeal metastases; (4) Vater’s ampullary carcinoma or biliary adenocarcinoma; (5) partial or complete intestinal obstruction; (6) a history of any other malignancy within five years, excepted: a) a consecutive 5-year disease free survival from single surgery of other malignancies or b) cured cutaneous basal cell carcinoma or cured in situ carcinoma of the cervix; (7) received major surgery within 4 weeks; 8) infected with HIV, hepatitis B or hepatitis C; (9) having serious concomitant diseases.

### Treatment

Using the standard 3 + 3 design, this trial consisted of four escalating dose levels (1350 mg, 1620 mg, 1890 mg and 2160 mg BID) and corresponding four cohorts (A, B, C, and D) of three patients, with three additional candidates for each cohort as necessary. Each cohort was treated at only one dose level, and allowed to continue if patients were receiving clinical benefit. The cohort A was orally administered the starting dose of K-001 twice daily for 4 weeks as a circle, then the subsequent cohorts (B, C and D) were treated respectively at the increasing dose levels that had been fixed in advance. Only after the observation of one dose level was completed, can the trial at next higher dose level be carried out. Two or more dose cohorts may not be administered simultaneously.

Based on previous study results, the starting dose was the maximum one of the previous study, therefore a conservative incremental percent was set up, which was lower than what the improved Fibonacci’s method recommended (cohorts, dose levels and increment percents exhibited in Table 1).

**Table 1:** Cohorts and dose levels

Cohort	A	B	C	D
Incremental percent	Starting dose	20%	17%	14%
Dose level	2700 mg/day (1350 mg BID)	3240 mg/day (1620 mg BID)	3780 mg/day (1890 mg BID)	4320 mg/day (2160 mg BID)
Number of patient	3	3	3	3

Adverse events (AEs) were graded using the National Cancer Institute Common Terminology Criteria for Adverse Events version 4.0 (NCI CTCAE v4.0) [29], and the relationship of AEs to the study drug was evaluated. Dose limiting toxicity (DLT) was defined as any grade 3 AE or above that was definitely, or probably related to K-001 administration. The maximum tolerated dose (MTD) was defined as the highest dose level at which ≤33% of patients experience DLT [30].

If no DLT was observed, the trial escalated to the next dose cohort. If one of the three patients experienced a DLT at a certain dose level, three more patients would be administered at the same level, and patients with DLT should immediately be discontinued with medication and withdrawn from the trial; if DLT no longer presented, the trial proceeded at the next upper dose level; if DLT was still observed, the trial was closed, and all patients were followed for safety at day 28 after closure of the trial. The flowchart of the trial was presented in Supplementary Figure S1.

### Assessm ent

Medical histories, disease characteristics and demographic data were collected at screening. The primary endpoints of this trial were safety and tolerability of the study drug, which were measured by adverse events (AEs),  vital sign, electrocardiogram, and laboratory tests at baseline and on days 8, 15, 22, 29 and 56. Tumor responses were assessed using Response Evaluation Criteria in Solid Tumors (RECIST version 1.0). Imaging studies (CT or MRI) of cancer sites were done within 2 weeks prior to the enrolment and on day 29. Clinical benefit responses were also estimated such as pain index by Numerical Rating Scale (NRS), and Quality of Life (QoL) by European Organization for Research and Treatment of Cancer Quality of Life questionnaire-Core 30 version 3.0 (EORTC QLQ-C30 3.0) at baseline and on days 8, 15, 22, 29 and 56 [31].

## Results

The trial was conducted in First People’s Hospital, School of Medicine, Shanghai Jiaotong University from February 2016 to December 2016, and the cutoff date for analysis was June 2017. The protocol was approved by the Institutional Review Board of the Hospital in accordance with the ethical principles of the Declaration of Helsinki (6th revision, 2008). All patients provided written informed consent for participation.

### Baseline characteristics of patients

A total of eighteen advanced PDAC patients were screened for this study, and six of them did not meet the inclusion criteria. Twelve eligible patients were analyzed in the study and two of them did not return on the day 56. The baseline characteristics are presented in Table 2. Median age of the twelve patients was 62 years (range, 53–67 years) and eight (67%) of them were male. Eight patients (67%) were on stage IV. Three patients (25.0%) did not receive any previous chemotherapy.

**Table 2:** Patient Demographics and Clinical Characteristics

	Number of patients (%)
Total	12 (100.0)
Median age in years (range)	62 (53–67)
Sex	
Male	8 (67.0)
Female	4 (33.0)
ECOG performance status	
0	0 (0)
1	12 (100.0)
Tumor stage at the time of diagnosis	
I	0 (0)
II	1 (8.3)
III	3 (25.0)
IV	8 (66.7)
Tumor stage at the time of enrolled	
Locally advanced PDAC	3 (25.0)
Metastatic PDAC	9 (75.0)
Metastasis site	
Liver metastasis	5 (55.5)
Lung metastasis	1 (8.3)
Peritoneal metastasis	2 (22.2)
Others	1 (8.3)
Prior chemotherapy therapy	
Yes	9 (75.0)
No	3 (25.0)

### Safety

During the dose escalation (1350, 1620, 1890 and 2160 mg twice daily), 12 patients completed the trial and were assessable for safety. Totally, 47 adverse events were observed, including 27 grade 1 AEs (57.4%), 17 grade 2 AEs (36.3%) and 3 grade 3 AEs (6.4%), and no grade 4 AE occurred. For the three grade 3 AEs, two of them were assessed as definitely not drug-related and the third one, gastrointestinal infection, as probably not drug-related, and all of them were reversible and manageable by treatments correspondingly. Among all 47 adverse events, 14 AEs were assessed as definitely or probably drug-related, with 8 grade 1 events (57.1%) and 6 grade 2 events (42.9%). These 14 AEs were reported in 7 patients (Table 3), with dyspepsia (21.4%) as the most frequent one, followed by flatulence, constipation, and haemorrhoids bleeding (above 10% of each). Besides, the correlation between the number of AEs and the dose levels was not significant (*p* = 0.334, 2-tailed), indicating that AEs were not dose dependent (AEs and dose levels exhibited in Table 4). In this phase I study, no DLT of oral K-001 with grade 3 or above drug-related AE was observed in patients during the escalating treatment cycles. Therefore, the MTD of K-001can be initially defined as 1350 mg-twice-daily for subsequent phase II/III studies. The safety of K-001 was quite good and might be suitable for patients of posterior line therapy, advanced age, and with poor performance status.

**Table 3:** Drug-related adverse events occurring in any patient (%)

Toxicity	Grade 1	Grade 2	Grade 3	Grade 4	Any Grade
Dyspepsia	1 (7.1)	2 (14.3)	0	0	3 (21.4)
Flatulence	1 (7.1)	1 (7.1)	0	0	2 (14.3)
Constipation	1 (7.1)	1 (7.1)	0	0	2 (14.3)
Haemorrhoids bleeding	1 (7.1)	1 (7.1)	0	0	2 (14.3)
Rash	0	1 (7.1)	0	0	1 (7.1)
ECG ST-T change	1 (7.1)	0	0	0	1 (7.1)
Dizzy	1 (7.1)	0	0	0	1 (7.1)
Diarrhea	1 (7.1)	0	0	0	1 (7.1)
Nausea	1 (7.1)	0	0	0	1 (7.1)
Total	8 (57.1)	6 (42.9)	0	0	14 (100.0)

**Table 4:** Dose levels and drug related AEs

Cohort	A	B	C	D	Total
grade 1 AE	0	2	2	4	8
grade 2 AE	1	1	4	0	6
Total	1	3	6	4	14

### Antitumor activity

According to the RECIST 1.0 criteria, all the 12 patients were evaluable for best overall response on day 29, and 10 patients were evaluable on day 56. Among the 12 patients, no patient presented complete response (CR) or partial response (PR), 10 patients (83.3%) maintained stable disease (SD), and 2 patients (16.7%) had progressive disease (PD). The objective response rate (ORR) was 0% and the 4-week disease control rate (DCR) was 83.3% (95% confidence interval [CI], 56.0–97.0%). The percent change in tumor size from baseline by dose cohort was shown in Table 5.

**Table 5:** Antitumor activity of K-001 in PDAC patients

Patient ID	Dose cohort (mg, BID)	Best overall response	Change in tumor size from baseline (%)	NRS			QoL			CRP (mg/L)			OS (days)
				Baseline	Day 29	Day 56	Baseline	Day 29	Day 56	Baseline	Day 29	Day 56	
1	1350	SD	0^b^	4	3	3	50	50	50	0.5	1.4	0.8	457
2	1350	SD	4.4^b^	5	8	8	47	54	54	5.4	49.4	62.7	167
3	1350	SD	4.3	8	8	4	74	68	85	33.6	8.0	67.2	114
4	1620	SD	9.4	0	3	NE	34	53	NE	64.2	88.5	NE	101
6	1620	SD	−2.9	5	8	7	56	67	73	3.4	13.5	3.6	378
7	1620	SD	0^b^	2	2	2	51	48	86	0.3	0.4	3.3	171
9	1890	SD	0^b^	2	4	3	31	28	30	3.7	1.2	7.9	759
10	1890	PD	−28.6^a^	4	4	NE	52	47	NE	5.0	21.8	NE	79
11	1890	PD	0^ab^	1	1	1	31	31	40	2.1	35.0	5.9	294
13	2160	SD	0^b^	5	5	5	57	58	59	0.1	2.8	0.3	441
16	2160	SD	2.2^b^	1	1	3	35	32	32	1.4	0.5	1.0	396
18	2160	SD	2.1^b^	4	3	4	54	51	50	0.8	0.9	6.6	166

*Abbreviations*: *SD* stable disease, *PD* progressive disease, *NE* not evaluable

^a^ Disease progression due to the appearance of new lesions

^b^ Showed the percent change of the of target lesion (PDAC) as compared with baseline

Changes of NRS score, QoL, and C-reactive protein (CRP) at baseline and on day 29, day 56 (2 patients not evaluable-16.7%) was also shown in Table 5. Compared to their baselines, NRS scores obtained relieved or stable in 8 patients (66.7%) on day 29, and in 6 patients (50.0%) on day 56, among which 3 patients did not need to take analgesics. QoL scores kept stable or improved in 6 patients (50.0%) on day 29, and in 7 patients (58.3%) on day 56. CRP levels were decreased or stable in 3 patients (25.0%) and increased in 9 patients (75.0%) on day 29, and decreased or stable in 1 patient (8.3%) and increased in 9 patients (75.0%). Furthermore, the results of paired samples t test (Table 6) indicated that on the whole, NRS, QoL, and CRP were increased on day 29 and day 56, compared to their baselines respectively, but did not reach the significant level. That is, NRS, QoL, and CRP remained stable during these periods. After the determination of DLT, the patients with stable disease further took K-001 orally. The AE and tumor response did not have a further follow-up. We followed-up the OS of the patients (The detailed date were presented in Table 5). The median OS was 171 days which were even longer than the 5-Fu/LV group (4.2 months) in NAPOLI-1 trial which was used for second lines therapy [26].

**Table 6:** Student *t* test of the paired samples

	Paired Differences	Mean	Std. Deviation	Std. Error Mean	95% Confidence Interval of the Difference		t	df	Sig. (2-tailed)
					Lower	Upper			
Pair 1	NRSb - NRSd29	−.750	1.545	.446	−1.732	.232	−1.682	11	.121
Pair 2	QoLb - QoLd29	−1.250	7.448	2.150	−5.982	3.482	−.581	11	.573
Pair 3	CRPb - CRPd29	−8.5750	18.5407	5.3522	−20.3552	3.2052	−1.602	11	.137
Pair 1	NRSb - NRSd56	−.300	1.947	.616	−1.692	1.092	−.487	9	.638
Pair 2	QoLb - QoLd56	−7.300	11.842	3.745	−15.771	1.171	−1.949	9	.083
Pair 3	CRPb - CRPd56	−10.8000	19.2091	6.0744	−24.5414	2.9414	−1.778	9	.109

*Abbreviations*: *NRSb* NRS baseline, *QoLb* QoL baseline, *CRPb* CRP baseline, *NRSd29* NRS day 29, *QoLd29* QoL day 29, *CRPd29* CRP day 29, *NRSd56* NRS day 56, *QoLd56* QoL day 56, *CRPd56* CRP day 56

## Discussion

At present, the recommended first-line treatments are mainly GEM, nab-paclitaxel, Capecitabine, S-1, 5-Fu/LV, and FOLFIRINOX [12, 15–19, 23–25]. But the proportion of those patients, who are sustainable to single drug chemotherapy, is low, and who are applicable to combined chemotherapy, is even lower. And for those with poor performance status, the treatment options are even few. Furthermore, quiet few PDAC patients could receive nanoliposomal irinotecan as second line treatment, and the third line treatments of PDAC are even deficiency [26]. Thus a safe, effective, and low toxic drug is urgently needed in PDAC.

Clinical studies in recent years have confirmed that the efficacy of FOLFIRINOX in advanced PDAC is significantly better than that of GEM. At the same time, however, the incidence of grade 3 or 4 neutropenia, febrile neutropenia, thrombocytopenia, diarrhea, and sensory neuropathy associated with the FOLFIRINOX was significantly higher than that associated with GEM. In addition, two patients even died of treatment-related factors [23]. The current study confirmed that K-001 is quite well tolerated in PDAC patients, and no dose limited toxicity (DLT) was observed in the treatment cycles. During the trial, the drug-related (definitely or probably) AEs were grade 1–2, mainly manifested as gastrointestinal reactions, and the symptoms were relieved or disappeared after appropriate treatments. All the AEs did not interfere with the dose escalation in the trial. The outcomes of the current study demonstrated that K-001 was very safe for PDAC patients. Furthermore, the safety of K-001 was much better than GEM and S-1 which was recommended for PDAC patients with poor performance status [15, 24].

**Fig. 1:**
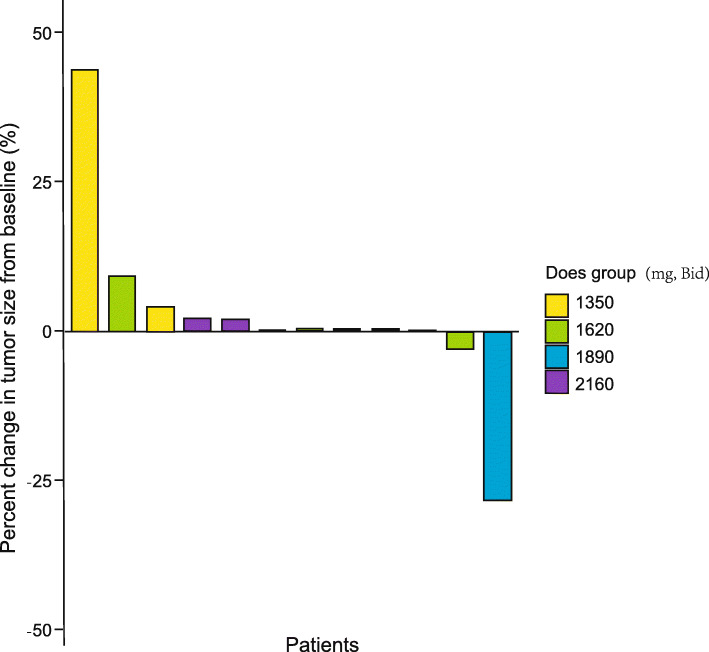
Waterfall plot of percent change in tumor size from baseline by dose group

Although efficacy was not the primary endpoint of the current trial, objective response rate (ORR), disease control rate (DCR), and other indicators of clinical benefit (NRS, QoL, and CRP) were evaluated to provide clues for subsequent phase II/III studies. In the current trial, the DCR of K-001 reached 83.3% (95% CI, 56.0–97.0%), with more than 80% of the enrolled patients exhibited either tumor shrinkage or stabilization according to the RECIST 1.0 criteria (Fig. 1). In the phase III study of FOLFIRINOX versus GEM, the DCR of FOLFIRINOX was 70.2% and of GEM was 50.9% [23]. In MPACT trial, the DCR of Nab-paclitaxel plus GEM was 48% and of GEM was 33% [25]. In GEST trial, the DCR of S-1 was 63.3%, of GEM was 62.7 and of GS was 71.5% [24]. Compared with the outcome indicators of the above studies, K-001 had a quite impressive DCR for advanced PDAC patients in this trial. Furthermore, the median OS of the trial was 171 days (5.7 months) and the patient with the longest survival was 759 days (25.3 months). All the enrolled patients were at posterior line therapy and the median OS of this trial was longer than the 5-Fu/LV group (4.2 months) in NAPOLI-1 trial which was used for second lines therapy [26].

Performance status (PS), measured by QoL in the trial, is one of the important prognosis indicators of PDAC patients. Poor PS is commonly accompanied with advanced PDAC patients, and results in limited options other than palliative systematic treatment [32]. Pancreatic cancer pain, measured by NRS, is associated with poor prognosis in PDAC, and is one of the main causes of decreased quality of life and survival [33]. CRP is an important aggressive marker of PDAC and its level is relevant to worse prognosis [34–36]. Paired samples t test indicated that during the trial, variations of NRS, QoL and CRP were not statistically significant, meaning that they maintained stable in the cycles of the trial. These outcomes are corresponding to DCR (SD) of 83.3%, and signify that K-001 may contribute to PS improvement, have certain analgesic effect, and influence CRP level.

## Conclusions

In the current phase I study, K-001 has demonstrated satisfactory safety and tolerability in the treatment of advanced PDAC patients, as well as meaningful antitumor activity in terms of DCR and clinical benefits. But no ORR was observed in this trial which indicated that K-001 might be more suitable for patients of posterior line therapy, advanced age, and with poor performance status. With the outcomes of the current trial, the MTD of K-001 can be initially defined as 1350 mg-twice-daily. Furthermore, a multicenter, randomized and double blind phase II/III studies of K-001 has been further carried out in PDAC patients after second line treatment (ChiCTR-IIR-17013424, Chinses Clinical Trial Registry).

## Supplementary Information

**Additional file 1.**

## Data Availability

The datasets used and/or analyzed during the current study are available from the corresponding authors on reasonable request.

## Abbreviations

PDAC: Pancreatic ductal adenocarcinoma
DLT: Dose-limiting toxicity
MTD: Maximum tolerated dose
RECIST: Response Evaluation Criteria in Solid Tumor
AE: Adverse event
SD: Stable disease
PD: Progressive disease
ORR: Objective response rate
DCR: Disease control rate
GEM: Gemcitabine
FOLFIRINOX: Irinotecan, oxaliplatin, and leucovorin-modulated fluorouracil
OS: Overall survival
PFS: Progression-free survival
PGN: Peptidoglycan
RD: Recommended dose
ECOG PS: Eastern Cooperative Oncology Group performance status
ULN: Upper limit of normal
ALT: Alanine aminotransferase
AST: Aspartate aminotransferase
NCI CTCAE: the National Cancer Institute Common Terminology Criteria for Adverse Events
NRS: Numerical Rating Scale
QoL: Quality of Life
EORTC QLQ-C30 3.0: European Organization for Research and Treatment of Cancer Quality of Life questionnaire-Core 30 version 3.0
CR: Complete response
PR: Partial response
CI: Confidence interval
CRP: C-reactive protein
