# Supplementary figures and images for: A phase I study of the safety and activity of K-001 in patients with advanced pancreatic ductal adenocarcinoma
Source: BMC Cancer. 2021 Jun 7;21:672. doi: 10.1186/s12885-021-08375-6 (PMC8183060; doi:10.1186/s12885-021-08375-6)

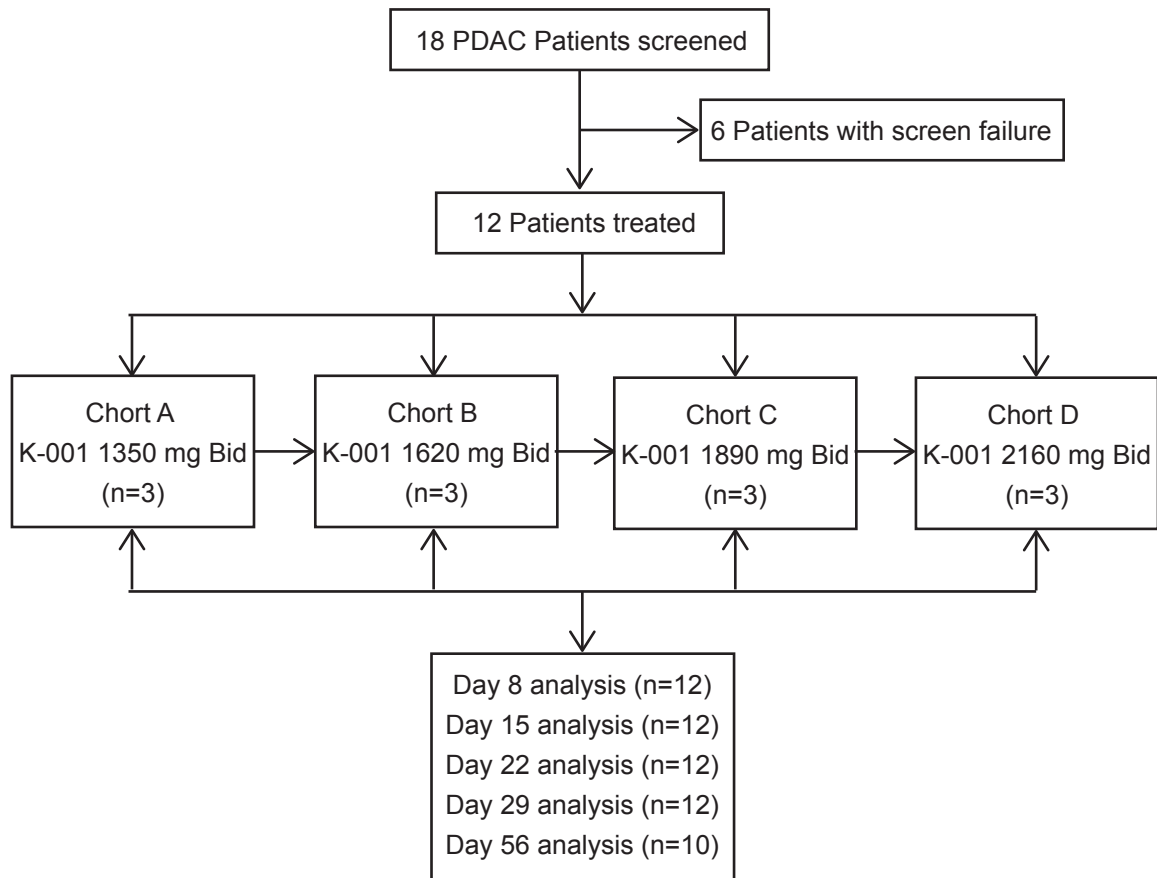

Supplement: Supplementary file 1 — Additional file 1. [file 12885_2021_8375_MOESM1_ESM.pdf]
